# Collision Mechanisms of Particles in the Al–Ti Plasma Plume Induced by Pulsed Laser Ablation

**DOI:** 10.3390/ma19091904

**Published:** 2026-05-06

**Authors:** Shimin Chang, Ruiqi Shen, Lizhi Wu

**Affiliations:** 1 School of Chemistry and Chemical Engineering, Nanjing University of Science and Technology, Nanjing 210094, China; 318103010119@njust.edu.cn (S.C.); rqshen@njust.edu.cn (R.S.); 2 Micro-Nano Energetic Devices Key Laboratory of MIIT, Nanjing 210094, China

**Keywords:** pulsed laser ablation, pulsed laser deposition, Al–Ti plume, background gas pressure, collision accumulation, species transport, plume propagation

## Abstract

The dynamics of pulsed laser ablation plumes strongly influence thin-film deposition quality; however, pressure-dependent collision accumulation and component-resolved transport in binary metal plumes remain poorly understood. In this study, a kinetic-statistical model was employed to investigate the propagation of an Al_0.75_Ti_0.25_ plume in a low-pressure inert Ar background at a laser fluence of 8 J/cm^2^. The results show that, at t = 0.56 μs, the cumulative number of particles that have experienced at least one collision increases with pressure in the range of 0.001–1 Pa and follows an approximately power-law dependence. Across the entire pressure range and throughout the 0.08–0.56 μs interval, the collision fraction of Ti remains consistently higher than that of Al. Based on a Ti-normalized cumulative collision index, the propagation regime can be classified into a near-free-flight region, a transition region, and a collision-influenced region, with only minor temporal variations in the corresponding boundary pressures. Further analysis of the initial velocity spectrum shows that Ti contributes more strongly to the high-velocity tail, which explains its greater propensity for collision during propagation. These findings provide a quantitative framework for understanding pressure-dependent collision accumulation and species transport in binary metal plumes under inert low-pressure conditions.

## 1. Introduction

Pulsed laser deposition (PLD) [1] and pulsed laser ablation (PLA) [2] have been widely used for the fabrication of metallic, oxide, and multicomponent thin films [3,4] because of their high instantaneous power density, non-contact energy delivery, and broad adaptability to different material systems [5]. Upon laser irradiation, a high-temperature plume composed of neutral atoms, ions, and clusters forms above the target surface within an extremely short timescale [6,7]. Its subsequent propagation is not simply inertial expansion but also involves interparticle collisions, energy redistribution, and scattering by the background gas [8]. Plasma dynamics determine not only the energy and spatial distributions of particles arriving at the substrate [6], but also the deposition flux, film uniformity, and subsequent microstructural evolution of the film [9,10]. Therefore, an accurate characterization of plasma propagation mechanisms is a crucial foundation for understanding the deposition process and optimizing process parameters [11]. This is particularly relevant for Ti-containing material systems, where pulsed-laser-ablation/plume dynamics are closely related to the fabrication and control of functional Ti-based nanostructures [12].

Among the many factors influencing plume propagation behavior, the background gas is one of the most critical external control parameters. Previous studies have shown that changes in background gas pressure and the corresponding number density can significantly alter the collision frequency, kinetic energy dissipation, and propagation range of plume particles [13], ultimately affecting the state of particles reaching the substrate [14]. For binary metal plumes such as Al–Ti, interspecies differences in atomic mass, velocity distribution, and energy distribution [15] give rise to distinct collision and transport behaviors [16], making their propagation mechanisms inherently more complex than those of single-component systems.

Regarding plume propagation, researchers have proposed classical analytical models such as shockwave [17], drag [18], and adiabatic thermalization [3]. However, these models are generally applicable only over limited pressure ranges and mainly describe plume-front evolution, making it difficult to fully resolve the internal composition, velocity structure, and spatial inhomogeneity of the plume [19,20]. Particularly in multicomponent systems, relying solely on the propagation laws of the plume front makes it challenging to establish a connection between background parameters, component differences, and transport mechanisms [21]. To overcome this limitation, Wijnands et al. [22] proposed a generalized numerical model applicable to PLD plume propagation. This model advances the plume expansion problem from a simple front description to statistical tracking of particle composition and velocity distribution within finite spatial elements. The model yielded results that were in good agreement with experimental observations in complex oxide systems, indicating that a modeling approach based on particle swarm statistics is more suitable for describing the propagation process of multicomponent plumes.

Nevertheless, previous studies have focused mainly on the propagation and reaction behavior of oxide plumes in reactive atmospheres [23,24,25], whereas a systematic numerical framework for collision accumulation, species-resolved transport, and regime classification in binary metal plumes under inert backgrounds is still lacking [14,26,27]. In particular, under dilute to transitional pressure conditions, how background pressure influences particle collision accumulation by regulating background number density [28] and further alters the transport dominance of different components remains to be thoroughly elucidated.

In this work, a kinetic-statistical modeling approach is used to investigate the propagation behavior of an Al_0.75_Ti_0.25_ plume in an Ar background at a fixed laser fluence of 8 J/cm^2^. In the present work, this value is not treated as a threshold-derived optimum fluence for the specific Al–Ti target but rather as a representative operating condition above the onset of stable plume generation and suitable for isolating the effect of background pressure within a univariate scan. This distinction is important because the relationship between the ablation threshold and the most efficient fluence has been discussed in both theoretical and experimental laser-ablation studies [29,30]. Unlike previous studies on the oxidation of TiO_2_/O_2_ systems, the present work does not consider chemical reactions but focuses on collision accumulation, compositional differences, and the identification of mechanism boundaries in an inert background. The “ever-collided” statistic is introduced to characterize the scale of the particle ensemble that has collided at least once, and a Ti-normalized cumulative collision index is proposed to construct a mechanism partition map. Combined with analysis of the initial velocity spectrum and the kinetic-energy contribution in the high-velocity range, this study reveals how background pressure regulates collision accumulation and transport dominance in Al–Ti binary plumes. This study contributes to a deeper understanding of the propagation behavior of binary metal plumes and provides a theoretical basis for parameter optimization and interpretation of transport-related effects in multicomponent metal-film deposition.

## 2. Numerical Model Foundation, Adaptation, and Data Definitions

### 2.1. Model Foundation and Scope

To describe the early-stage transport and collision evolution of an Al–Ti ablation plume in an inert Ar atmosphere, a kinetic-statistical framework is adopted. The model draws upon the generalized numerical treatment of pulsed laser deposition plume propagation proposed by Wijnands et al. [22]. Wijnands et al. [22] explicitly discussed the limitations of traditional analytical models. These analytical descriptions are typically applicable only within a limited pressure range and primarily describe the motion of the plume front, failing to provide sufficient information regarding the internal composition, velocity distribution, and component-resolved transport behavior of the plume. In contrast, the generalized numerical framework can track the evolution of composition and velocity distributions for different particle populations within a finite spatial domain, thus providing a more suitable basis for the study of multicomponent plumes.

Building on this foundation, this paper extends the aforementioned modeling approach from the TiO_2_/O_2_ system to the Al–Ti/Ar binary metal-inert gas system, while explicitly redefining the research objectives and output variables. This study does not consider oxidation reactions but focuses on the control mechanisms of background pressure on collision accumulation, component-resolved transport, and mechanism boundaries in an inert atmosphere. Therefore, this study retains the core framework of such models regarding “statistical transport of multicomponent particle populations” while removing reaction pathways and enhancing the treatment of initial velocity spectra, collision exposure, post-collision velocity updates, and component-resolved statistical quantities.

Specifically, the present model is designed to achieve the following three objectives:

(1) Construct initial velocity distributions with thermal statistical consistency for the Al and Ti components under the constraint of post-ablation energy distribution;
(2) Simulate the propagation and collision evolution of plume particles and background Ar particles within a discrete space-time framework;
(3) To extract quantitative relationships between background pressure, collision accumulation, and compositional dominance based on repeatable statistical definitions, and to further establish a mechanism partition map.

Unlike analytical models that focus solely on the plume front position, this study focuses on the collision participation and statistical structure of multicomponent particles during transport; therefore, a numerical description based on particle distribution functions and spatial partitioning is more appropriate.

To isolate the effect of environmental control factors and minimize interference from multivariable coupling, a univariate scanning strategy is adopted: the laser fluence is fixed at 8 J/cm^2^, the atomic ratio of the target material composition is fixed at Al_0.75_Ti_0.25_, the background gas is selected as Ar, and only the background pressure P_bg is varied. Unless otherwise specified, all statistical quantities discussed in the Methods and Results sections of this paper are defined and analyzed with respect to the axial direction θ = 0°. This approach is consistent with the concept of a “representative cross-section at a specific angular direction” in the three-dimensional plume statistics framework, i.e., under the approximation that net exchange between angular bins is negligible, axial statistics can effectively characterize transport behavior along the primary propagation direction.

The numerical program was implemented in Mathematica and executed on a high-performance computing cluster using batch jobs submitted via the PBS/Slurm scheduling system. Each compute node had dual Intel Xeon 6132 processors (2.6 GHz, 28 cores per node) and 192 GB memory.

In the present work, a fixed laser fluence of 8 J/cm^2^ was adopted to isolate the effect of background pressure within a univariate scan. This value is not treated here as a threshold-derived optimum fluence for the Al_0.75_Ti_0.25_ target but rather as a representative operating condition above the onset of stable plume generation and suitable for examining pressure-controlled collision accumulation and component-resolved transport. If a different fluence, especially a threshold-optimized fluence, were used, the initial velocity spectrum, the relative weight of the high-velocity tail, and the absolute cumulative collision counts could change, although the Ti-normalized mechanism-identification framework developed here would remain applicable.

### 2.2. Initialization of Ablation Particles

#### 2.2.1. Total Number of Ablation Particles

Based on the geometric-density estimation approach described in the model documentation, the total number of particles ablated by a single pulse is determined by the ablated volume and the number of structural units in the material. Assuming the ablation spot is a square region with area $A_{spot}$ and the ablation depth is $d_{abl}$ (treated as a constant in the configuration shown), the ablated volume is

(1) $$V_{abl}=A_{spot}d_{abl}$$

Let the unit cell volume of the material be $V_{cell}$ (for multi-component materials, the component-weighted effective unit cell volume may be used); then the number of ablated unit cells is

(2) $$N_{cell}=\frac{V_{abl}}{V_{cell}}$$

If each unit cell contains $n_{cell}$ atoms, then the total number of ablated atoms (total number of plume particles) is

(3) $$N_{abl}=N_{cell}n_{cell}$$

For binary alloys, the number of particles is distributed according to the atomic ratio. For Al_0.75_Ti_0.25_: $N_{Al}={0.75N}_{abl}$, $N_{Ti}={0.25N}_{abl}$.

It should be noted that this initialization does not explicitly simulate complex ablation mechanisms such as phase explosion or molten ejection; instead, it provides statistically consistent plume source terms, thereby allowing the analysis to focus on transport and collision-accumulation behavior.

#### 2.2.2. Energy Balance and RMS Velocity

The model employs the principle of energy balance to determine the initial velocity scales of each component. Let the energy absorbed per unit pulse be $E_{abs}$. A portion of this energy is used for “activation costs” such as unit cell dissociation and bond breaking, while the remaining energy is allocated to particle kinetic energy (as described by the model, the explicit contribution of electronic excitation energy to energy conservation is neglected for simplicity). For a component $x\in \left\{Al,\;Ti\right\}$, the total energy allocated to its kinetic energy is denoted as $E_{k,x}$, and the average kinetic energy of the component is

(4) $${\bar{\varepsilon }}_{k,x}=\frac{E_{k,x}}{N_{x}}$$

The corresponding root-mean-square velocity is

(5) $$v_{rms,x}=\sqrt{\frac{2{\bar{\varepsilon }}_{k,x}}{m_{x}}}$$

where $m_{x}$ is the mass of the component particle. This RMS velocity is used as the scaling parameter for subsequent sampling of the initial velocity distribution.

#### 2.2.3. Maxwell–Boltzmann Initial Velocity Distribution

To ensure thermodynamic consistency, the initial velocity distribution is described by a Maxwell–Boltzmann (MB) form rather than by an empirical Gaussian approximation. Therefore, the initial velocity distribution for component x is taken as

(6) $$f_{x}\left(v\right)=4\pi {\left(\frac{m_{x}}{2\pi k_{B}T_{x}}\right)}^{3/2}v^{2}\exp\left(-\frac{m_{x}v^{2}}{2k_{B}T_{x}}\right)$$

where $T_{x}$ is the effective temperature corresponding to the average kinetic energy of the component, and satisfies the energy equipartition relation:

(7) $$\frac{3}{2}k_{B}T_{x}={\bar{\varepsilon }}_{k,x}$$

This form naturally reflects the effect of mass differences on the broadening of the distribution: lighter elements have a broader distribution, while heavier elements have a narrower one, consistent with the qualitative explanation based on Graham’s law as described in the model.

### 2.3. Initialization of the Background Ar

#### 2.3.1. Background Temperature and Number Density

The background gas temperature is assumed to be uniform throughout the space, $T_{bg}=300\;K$. Although temperature gradients may exist near the target surface in reality, this paper does not introduce this complexity in order to highlight the pressure effects and keep the model simple. The background number density is given by the ideal gas equation of state:

(8) $$n_{bg}=\frac{P_{bg}}{k_{B}T_{bg}}$$

Ar particles are uniformly distributed in space.

#### 2.3.2. Initialize Background Speed

Because the thermal velocity of the background gas at room temperature (on the order of 10^2^ m/s) is much smaller than the typical velocity of plume particles (10^3^–10^4^ m/s), and because the background gas has no preferred direction of motion, its mean initial velocity is approximated as zero. To ensure consistency in the pressure scan, this assumption is maintained throughout this paper.

#### 2.3.3. Model Scope and Simplifying Assumptions

The present model is designed to isolate the pressure-controlled collision accumulation behavior of a binary Al–Ti plume in an inert low-pressure Ar background over the 0.08–0.56 μs time window. For this reason, several simplifying assumptions are adopted. First, only plume–background collisions are explicitly retained, because background-gas-induced momentum exchange is the primary external mechanism governing the cumulative collision statistics studied here. Second, inter-plume collisions are not explicitly resolved, since the current work focuses on pressure-controlled component-resolved transport rather than dense near-target plume equilibration. Third, electronic excitation energy is neglected in the energy-balance step because the aim is to construct thermally consistent initial velocity distributions rather than to model detailed internal-state kinetics. Finally, the background gas is treated as spatially isothermal at 300 K, and local temperature gradients near the target are not considered. These assumptions allow the dominant pressure-dependent transport and collision trends to be extracted clearly within the scope of the present study.

### 2.4. Discrete, Propulsion, and Collision Handling

#### 2.4.1. Distance-Based Binning and Physical Resolution

Along a given observation direction (in this paper, θ = 0°), the space is discretized into bins with a physical width of ∆r = 5 μm. At each time step t, the component counts are output and accumulated within each bin; subsequently, the bins are summed to obtain the global statistics. Therefore, $\sum_{r}\left(\cdot \right)$ can be viewed as a one-dimensional discrete integral approximation along the direction of propagation.

#### 2.4.2. Collision Categories and Collision Estimation Within Bins

According to the model description, collisions between plume particles and background Ar are treated as head-on, perfectly elastic collisions. For a plume component x (with velocity $v_{x}$) propagating through a distance bin of length ∆r, its collision exposure with the background gas can be described using a collision rate framework. Let $n_{bg}(r)$ denote the background number density (assumed uniform in this paper), and $\sigma_{bg}$ denote the effective scattering cross section between component x and the background gas g(Ar). In the present work, the effective scattering cross section is estimated using the hard-sphere geometric approximation, $\sigma_{xg}=\pi {\left(r_{x}+r_{g}\right)}^{2}$. Where $r_{x}$ is the atomic radius of species x and $r_{g}$ is the atomic radius of Ar. Accordingly, separate values are used for Al–Ar and Ti–Ar collisions.

Based on the concept of “collision path volume,” the collision exposure scale within a bin is proportional to $n_{bg}\sigma_{bg}∆r$. To account for the effect of relative motion on the collision probability, a relative velocity factor is introduced:

(9) $$v_{rel}=\left|v_{x}-v_{g}\right|$$

In the near-zero background velocity approximation, $v_{g}\approx 0$. Therefore, the effective collision rate can be written as

(10) $$u_{xg}\left(r\right)\propto n_{bg}\left(r\right)\sigma_{xg}v_{rel}$$

where the cumulative ever-collided particle number varies approximately proportionally with $\sigma_{xg}$ to first order. To enable component-sensitive mechanism identification, a Ti-normalized cumulative collision index is introduced, as formally defined in Equation (16). Equation (16) is normalized by the 1 Pa value at the same time, so a substantial part of the multiplicative effect of $\sigma_{xg}$ cancels out. Therefore, moderate variations in σ primarily affect the absolute collision counts, whereas the corresponding mechanism-boundary pressures $P_{1}$ and $P_{2}$ remain comparatively robust.

This formulation reflects two primary controls:

(i) pressure dependence through the background number density: $n_{bg}\propto P_{bg}$;
(ii) enhanced weighting of high-velocity particles through the relative-velocity term ($v_{rel}$).

#### 2.4.3. Post-Collision Velocity Update

When a feather particle (mass $m_{x}$, pre-collision velocity $v_{x}$) undergoes a one-dimensional head-on elastic collision with a background Ar particle (mass $m_{g}$, pre-collision velocity $v_{g}$), the post-collision velocities ($v_{x}^{\prime }$, $v_{2}^{\prime }$) are given by the laws of conservation of momentum and kinetic energy:

(11) $$v_{x}^{\prime }=\frac{\left(m_{x}-m_{g}\right)v_{x}+2m_{g}v_{g}}{m_{x}+m_{g}},\;\;\;\;v_{2}^{\prime }=\frac{\left(m_{g}-m_{x}\right)v_{g}+2m_{x}v_{x}}{m_{x}+m_{g}}$$

Within a single time step, the displacement of colliding particles can be updated using a weighted average of their velocities before and after the collision, consistent with the time-step averaging approach described in the model.

### 2.5. Collision Statistics: ≥1 Instance of “Ever-Collided”

#### Ever-Collided Indicator Variable

The collision statistics used in this study are based on an “ever-collided” criterion: a particle is counted as collided if it has experienced at least one collision before the current time t, regardless of the total number of collisions. Define the indicator for particle i:

(12) $$X_{i}\left(t\right)=\left\{\begin{array}{c} 1,If\;particle\;i\;has\;collided\;at\;least\;once\;before\;time\;t\\ 0,No\;collision\;occurred \end{array}\right.$$

For species s within the distance bin:

(13) $$N_{coll}^{\left(s\right)}\left(t,r\right)=\sum_{i\in s\;\&\;r}X_{i}\left(t\right)$$

The total number of particles that have collided in the entire region is

(14) $$N_{collided}^{\left(s\right)}\left(t;P\right)=\sum_{r}N_{coll}^{\left(s\right)}\left(t,r\right)$$

The collision ratio is

(15) $$f_{coll}^{\left(s\right)}\left(t;P\right)=\frac{\sum_{r}N_{coll}^{\left(s\right)}\left(t,r\right)}{\sum_{r}N^{\left(s\right)}\left(t,r\right)}$$

It is important to note that $N_{collided}^{\left(s\right)}$ is a statistical measure of the set of particles that have collided “at least once,” not the “total number of collision events.”

### 2.6. Ti Criterion Mechanism Partitioning Index

To establish a practical regime map along the pressure axis, a Ti-normalized cumulative collision metric is defined as follows:

(16) $$\Phi_{Ti}\left(P,t\right)=\frac{N_{collided}^{Ti}\left(t;P\right)}{N_{collided}^{Ti}\left(t;1Pa\right)}$$

Based on the threshold, the pressure range is divided into: the near-free-flight region [7,31], where $\Phi_{Ti}<0.1$; the transition region [7,31,32], where $0.1\leq \Phi_{Ti}\leq 0.5$; and the impact region [7,32], where $\Phi_{Ti}>0.5$.

The corresponding mechanism boundary pressures $P_{1}(t)$ and $P_{2}(t)$ are obtained through interpolation in logarithmic coordinates. Since these values are normalized to 1 Pa at any given time, this significantly reduces the impact of absolute-scale uncertainties on the partitioning results, thereby establishing a component-sensitive and reproducible framework for mechanism identification.

### 2.7. Initial Velocity Spectrum and High-Velocity Range Indicators

For the same energy density of 8 J/cm^2^, analyze the initial velocity distributions $N_{Ti}(v)$ and $N_{Al}(v)$ for Ti and Al, respectively. Calculate the kinetic energy contribution for each velocity bin:

(17) $$E_{bin}^{\left(s\right)}\left(v\right)=\frac{1}{2}m_{s}v^{2}N_{s}\left(v\right)$$

Define two types of threshold velocities: the number-crossing velocity $v_{N}=min\left\{v:N_{Ti}(v)>N_{Al}(v)\right\}$; and the energy-crossing velocity $v_{E}=min\left\{v:E_{bin}^{Ti}(v)>E_{bin}^{Al}(v)\right\}$. Define the high-velocity tail as ${v>v}_{N}$, calculate the proportion of the tail in terms of both number and kinetic energy, and use these to explain why Ti collisions contribute more significantly to the source terms in subsequent pressure scans.

The primary operating conditions and key parameters mentioned in the text are shown in Table 1. Detailed definitions of the raw output columns and the derived statistical quantities used throughout the present study are provided in Table S1 of the Supplementary Materials.

## 3. Results

### 3.1. The Pressure–Volume Law Based on the Cumulative Number of Particles Involved in Collisions

**Figure 1:**
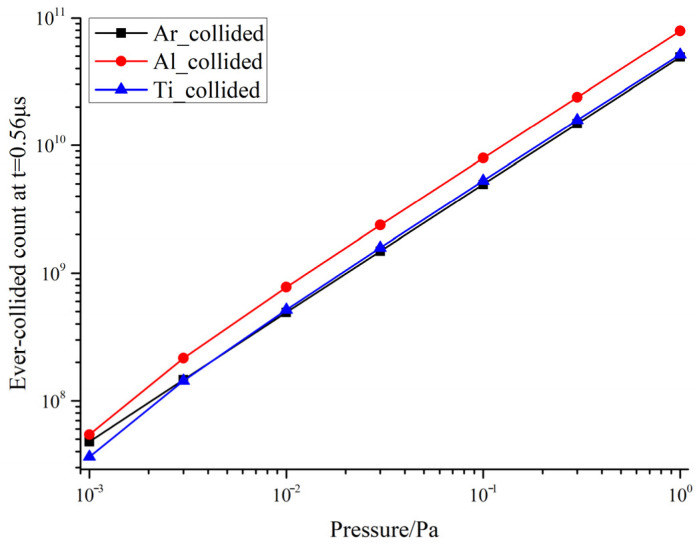
Log-log plot of the cumulative number of particles that have collided, $N_{collided}^{\left(s\right)}$, as a function of bulk pressure, $P_{bg}$, at t = 0.56 μs (θ = 0°).

Figure 1 shows the variation in the cumulative number of particles that have undergone collisions, $N_{collided}^{\left(s\right)}\left(t;P\right)$, for Ar, Al, and Ti species as a function of pressure under conditions of t = 0.56 μs and θ = 0°. The results indicate that the data are well described by a power-law relationship over the pressure range of 0.001–1 Pa:

(18) $$N_{collided}^{\left(s\right)}\left(P\right)\sim P^{\alpha_{s}}$$

As shown in Table 2, the full power-law fitting parameters for Ar, Al, and Ti are now provided over the entire 0.08–0.56 μs window, including the fitted exponent $\alpha_{s}$, the logarithmic prefactor $\log_{10}C_{s}$, and the fitting quality $R^{2}$. The results show that the fitted exponent for Ar remains extremely close to unity throughout the whole time range, while the exponents for Al and Ti remain very close at all observation times and gradually converge toward unity as plume propagation proceeds. More importantly, $\alpha_{Al}$ and $\alpha_{Ti}$ remain very close at all observation times, indicating that the two components follow essentially the same pressure-scaling behavior for cumulative collision accumulation. This result confirms that, under fixed laser fluence and composition conditions, background pressure is the dominant external parameter governing the cumulative increase in collision occurrence. This finding is consistent with previous studies showing that background gas enhances plume deceleration, deflection, and energy redistribution. Kántor and Szörényi [33] pointed out that elastic collisions in the background gas significantly alter particle transport and deposition profiles, while Harilal et al. [34] also demonstrated that plume propagation in an Ar background becomes markedly more complex due to collision enhancement. In contrast, the present study further quantifies this background effect in terms of the cumulative number of ever-collided particles, thereby providing a direct statistical basis for subsequent mechanism partitioning.

### 3.2. Differences in Collision Ratios and Composition (Ti and Al)

**Figure 2:**
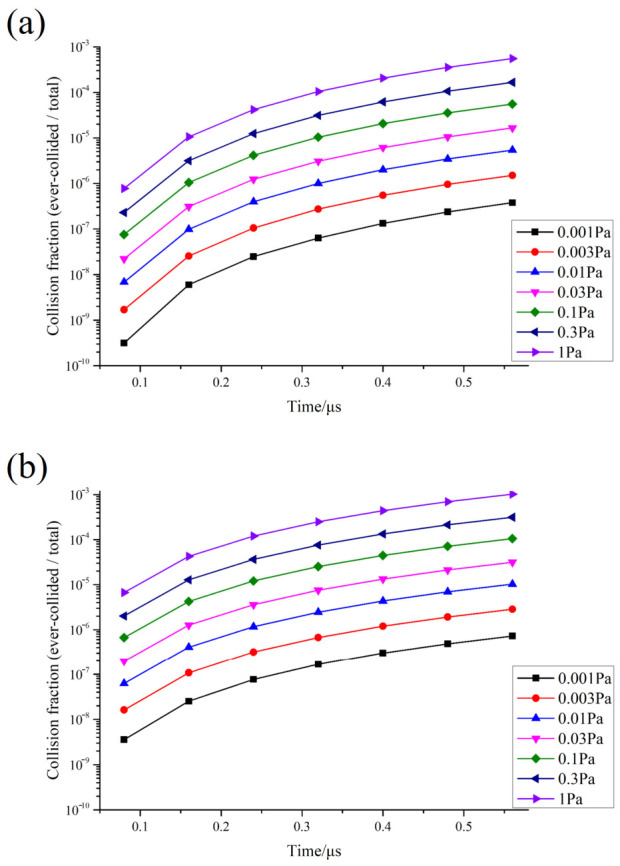
Evolution of the Al/Ti collision fraction $f_{coll}\left(t\right)$ over time at various pressures (log scale on the vertical axis): (**a**) Al; (**b**) Ti.

Figure 2 compares the collision ratios $f_{coll}^{Ti}\left(t;P\right)$ of Ti and Al across the full pressure range and the full time window. The results show that the collision ratios of both components increase monotonically with time (Table 3), reflecting the cumulative nature of the ever-collided particle ensemble during propagation. More importantly, for all pressures and at all observation times, the collision ratio of Ti is consistently higher than that of Al:

(19) $$f_{coll}^{Ti}\left(t;P\right)>f_{coll}^{Al}\left(t;P\right)$$

The complete time-resolved collision-fraction datasets for Ar, Al, and Ti at all investigated pressures are listed in Table S3 of the Supplementary Materials.

These results indicate that the response of the binary plume to the same collisional background is species dependent, with Ti exhibiting a consistently higher collision fraction during propagation. To examine the temporal robustness of this conclusion, this study further compared the collision ratios of Ti and Al at multiple observation times, and the results show that the statistical dominance of Ti remains consistent. This indicates that the Ti dominance observed here is not an artifact of a single sampling time, but rather a persistent feature of Al–Ti plume propagation in an Ar background. Research by Ojeda-G-P et al. [35] on multicomponent PLD indicates that background pressure and elemental mass ratios jointly influence the transport and compositional conservation of different components. This temporal consistency further supports the rationale for selecting Ti as the normalization reference species in subsequent mechanistic partitioning.

At the present stage, the present model is intended to establish a pressure-controlled collision-statistical framework rather than to reproduce a one-to-one experimental dataset for Al–Ti plumes. Nevertheless, the most suitable primary experimental benchmark would be time-resolved plume imaging under different background pressures, such as shadowgraphy, schlieren imaging, or gated fast imaging, because these methods can directly reflect pressure-dependent changes in plume spreading, front evolution, and propagation morphology. A complementary benchmark would be species-resolved time-resolved spectroscopy for comparing the relative transport persistence and spatial redistribution of Al and Ti. If a more direct validation of the high-velocity-tail interpretation is desired, velocity-sensitive diagnostics such as time-of-flight measurements would provide the most suitable benchmark for assessing the source-term differences predicted by the model.

### 3.3. Ti-Criterion Mechanism Phase Diagram and Quasi-Steady-State Mechanism Boundary Pressure

Based on the Ti-normalized index $\Phi_{Ti}\left(P,t\right)$ (Table 4), the mechanism transition pressure was obtained via logarithmic interpolation at t = 0.56 μs:

(20) $$P_{1}\approx 0.097\;Pa\left(\Phi_{Ti}=0.1\right),\;P_{2}\approx 0.494\;Pa\left(\Phi_{Ti}=0.5\right)$$

Therefore, within the study range of 0.001–1 Pa: P<0.10 Pa falls within the near-free-flight region; 0.10≤P≤0.50 Pa constitutes the transition region; and P>0.50 Pa falls within the collision-influenced region.

**Figure 3:**
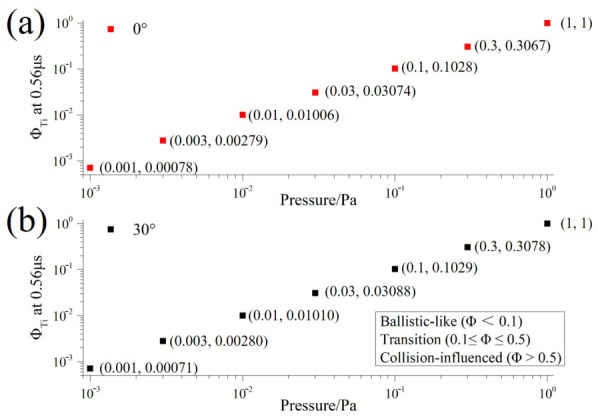
Mechanism diagram based on $\Phi_{Ti}$ at t = 0.56 μs: (**a**) θ = 0°; (**b**) θ = 30°.

Figure 3 maps the degree of collision accumulation under different pressure conditions to a unified scale using the Ti-normalized cumulative collision index $\Phi_{Ti}$, thereby enabling a quantitative classification of plume transport mechanisms. As the background pressure increases, the background particle number density rises, and the effective collision probability of particles along their propagation paths significantly increases; this is consistent with the definition of the collision rate in the model, which is based on background density, scattering cross-section, and relative velocity. When $\Phi_{Ti}<0.1$, the system exhibits behavior dominated by near-free flight; when $0.1\leq \Phi_{Ti}\leq 0.5$, it enters a transition region jointly controlled by free flight and collision dissipation; and when $\Phi_{Ti}>0.5$, collision effects become the dominant factor determining Ti transport behavior. Figure 3 is significant because it transforms the pressure dependence of plume propagation from an empirical description into a quantitative framework with clear boundaries and reproducible criteria.

At θ = 0°, the mechanism boundaries at t = 0.56 μs divide the pressure axis into a near-free-flight region, a transition region, and a collision-influenced region. The corresponding off-axis result at θ = 30° shows the same qualitative partitioning trend. Although the absolute magnitude of the collision statistics is reduced away from the centerline, the pressure dependence of the Ti-normalized cumulative collision index remains essentially unchanged. This indicates that the pressure-controlled mechanism transition identified in the present work is not an artifact of the axial direction alone but remains robust under moderate angular deviation. Therefore, the use of axial statistics as the primary reference is reasonable, while the comparison at 30° further supports the representativeness of the proposed mechanism-partition framework. The comparison between Figure 3a,b suggests that angular deviation mainly affects the absolute collision exposure rather than the normalized regime-identification criterion itself. In other words, moving from the centerline to an off-axis direction weakens the total collisional participation, but does not fundamentally alter the pressure-controlled transition from weakly perturbed transport to collision-influenced propagation. This angular consistency strengthens the physical significance of $\Phi_{Ti}$ as a component-sensitive mechanism indicator.

**Figure 4:**
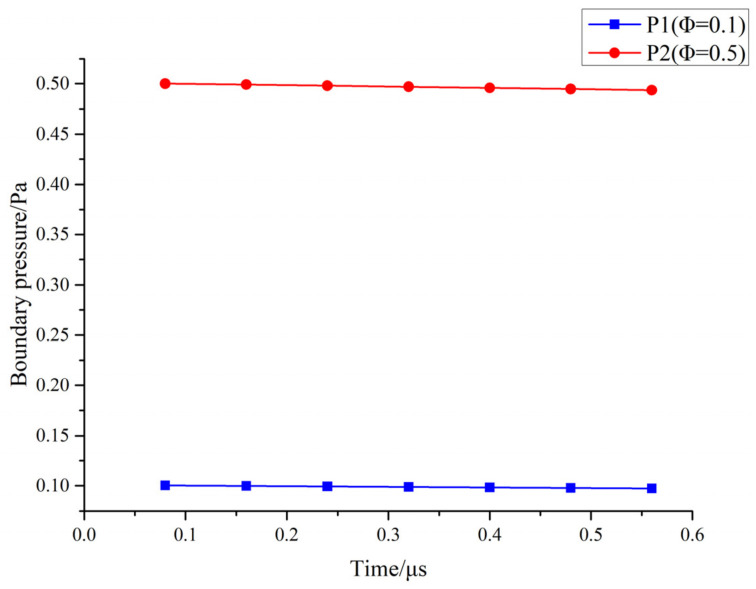
Time evolution of $P_{1}\left(t\right)$ and $P_{2}\left(t\right)$ within the range of 0.08–0.56 μs.

In Figure 4, the time window is further expanded to the full range (0.08–0.56 μs), revealing that the drift of $P_{1}\left(t\right)$ and $P_{2}\left(t\right)$ is minimal: $P_{1}$ decreases slowly from approximately 0.100 Pa to 0.097 Pa (about 3%), and $P_{2}$ decreases from approximately 0.500 Pa to 0.494 Pa (about 1%). This indicates that the regime boundaries are quasi-stationary within this time window and are governed primarily by pressure rather than by the specific sampling time.

Unlike traditional descriptions that rely on plume front displacement, the partitioning diagram presented in this paper is directly based on component-resolved collision participation, making it more suitable for identifying when background collisions transition from being a weak perturbation to becoming the dominant transport mechanism. Wijnands et al. [22] pointed out that classical models such as shockwave, drag, and adiabatic thermalization primarily focus on the plume front and struggle to capture the evolution of internal components and velocity distributions within the plume; therefore, statistical/numerical frameworks are more suitable for multicomponent systems. The mechanism partition map constructed in this paper represents a further extension of this approach to the Al–Ti/Ar binary system.

### 3.4. Quantity-Spanning Features in the Initial Velocity Spectrum

**Figure 5:**
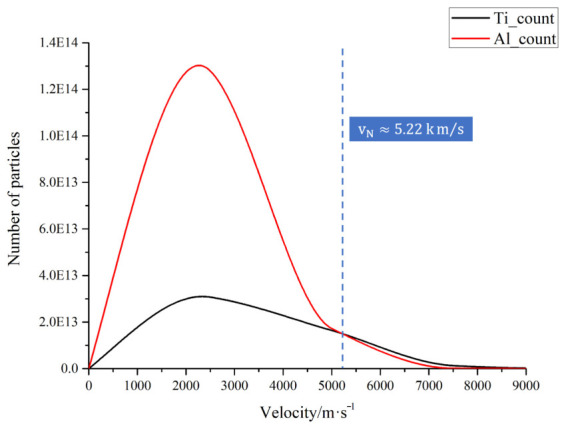
Velocity distributions $N_{Ti}\left(v\right)$ and $N_{Al}\left(v\right)$ for Ti and Al at t = 0.

To illustrate why Ti exhibits higher collision participation in subsequent propagation, Figure 5 returns to the t = 0 time point to compare the initial velocity distributions of Ti and Al, and defines a number-crossing velocity to identify the velocity range in which Ti begins to exceed Al in terms of the number of particles in a single bin. The number-crossing velocity is $v_{N}\approx 5.22km/s$; beyond this velocity, the number of Ti particles in that velocity bin exceeds that of Al. The results indicate that although Al is more abundant overall, Ti attains a local number advantage in sufficiently high velocity intervals.

This indicates that it is not the total number of components itself that determines the differences in subsequent collisions, but rather the local distribution structure within the high-velocity region. Because high-velocity particles traverse a larger effective distance during the early stage of propagation, this tail-region number advantage is translated into a greater likelihood of collision. Wijnands et al. [22], in their study of multi-component plumes, similarly emphasized that the initial velocity distribution of different elements significantly influences subsequent propagation behavior. The significance of Figure 5 in this paper lies in explicitly quantifying these source term differences and establishing a connection with subsequent collision statistics.

This indicates that even though Ti is less abundant than Al in total number, the high-velocity tail still gives Ti greater weight in terms of kinetic energy and collision effectiveness, thereby providing a source-term-level explanation for Ti’s higher collision fraction and the Ti-criterion mechanism partitioning.

### 3.5. The Initial Kinetic Energy Advantage of Ti in the High-Energy Tail

**Figure 6:**
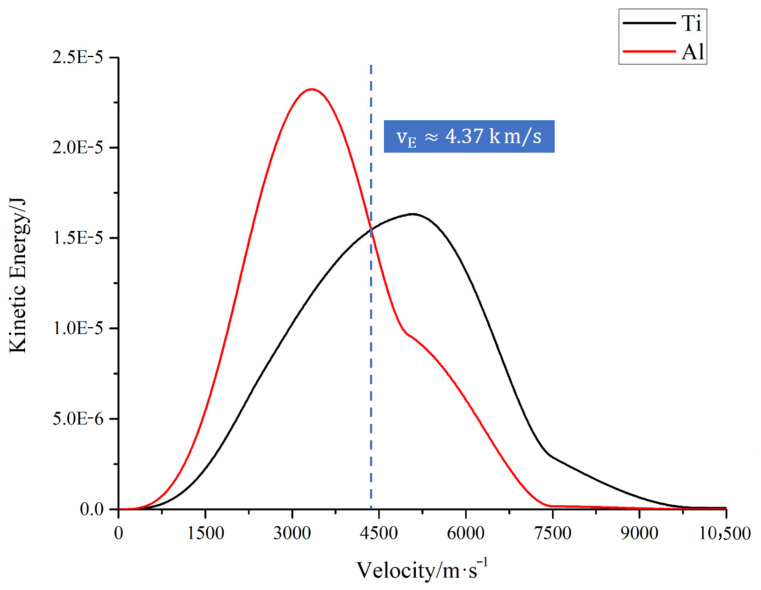
Kinetic energy contributions of $N_{Ti}\left(v\right)$ and $N_{Al}\left(v\right)$ at t = 0.

Figure 6 further compares the kinetic energy contributions of Ti and Al in velocity bins at time t = 0, and defines the energy-crossing velocity. The energy-crossing velocity $v_{E}\approx 4.37km/s$: above this velocity, the kinetic energy contribution of Ti in that velocity bin exceeds that of Al. The results show that although the high-velocity tail contains a limited number of particles, it accounts for a significantly higher proportion of the total kinetic energy, with Ti contributing the majority of this tail energy. This indicates that Ti dominance is reflected not only in its local abundance within the high-velocity region, but also in its disproportionate contribution to the kinetic energy that drives early propagation and collision exposure. A summary of the key statistical indicators of the initial velocity spectrum, including the crossover velocities and high-velocity-tail fractions, is provided in Table S2 of the Supplementary Materials.

Defining the high-velocity tail as ${v\geq v}_{N}$ yields the typical “few high-energy” characteristic: particles in the high-velocity tail account for only about 5.75% of the total particle count; however, they contribute as much as approximately 25.9% of the total kinetic energy, with Ti accounting for about 72% of the tail’s kinetic energy. Therefore, the fact that Ti consistently exhibits a higher collision fraction during subsequent propagation is not solely due to differences in mass or total number, but rather stems from the combined effects of the initial velocity spectrum, the high-velocity region, and the distribution of kinetic energy. The discussion by Kántor and Szörényi [33] regarding the influence of the mass ratio on background transport, as well as the results on multi-component differential transport by Ojeda-G-P et al. [35], indirectly support this interpretation. A further contribution of this paper lies in tracing the ultimate transport differences back to the energy structure of the initial high-velocity region.

## 4. Discussion

A comprehensive analysis of Figure 1, Figure 2, Figure 3, Figure 4, Figure 5 and Figure 6 reveals that the background pressure determines the intensity of the collision environment in which the plume resides, as evidenced by the fact that the cumulative number of colliding particles increases approximately in a power-law relationship with pressure; however, different components respond differently to this environment, with Ti exhibiting a consistently higher collision participation rate than Al throughout the entire time window. Further analysis indicates that this compositional difference is not a random phenomenon during propagation, but is determined at time t = 0 by the initial velocity spectrum and the distribution of kinetic energy in the high-velocity range. Based on this, this study uses the Ti-normalized cumulative collision metric to transform the pressure axis into the near-free-flight region, the transition region, and the collision-influenced region, thereby achieving an advancement from the description of statistical laws to the identification of mechanistic boundaries.

From the viewpoint of thin-film growth, the transport asymmetry between Al and Ti suggests that background pressure may influence not only the total arriving flux but also the spatial composition distribution on the substrate. Because Ti exhibits a consistently higher collision fraction and stronger weighting in the initial high-velocity tail, it is expected to undergo stronger pressure-induced redistribution during propagation. This implies that the component ratio reaching the substrate centerline and off-axis regions may evolve differently with pressure, which is relevant to film stoichiometry and in-plane uniformity. At the present stage, the model provides a mechanistic indication of this possibility rather than a fully resolved substrate-composition map. Nevertheless, it establishes a physically grounded basis for future extensions toward deposition-profile and composition-uniformity prediction.

The present pressure scan was intentionally simplified to isolate the effect of background pressure. In future work, the Ti-normalized collision framework may be extended to reactive backgrounds by introducing reaction pathways and pressure-dependent chemical transformation terms, and to ternary or multicomponent alloy plumes by defining analogous component-normalized collision indices. This would allow the present mechanism-partitioning strategy to be applied to more complex plume systems.

Overall, the results of this study are consistent with existing research conclusions regarding background gas-enhanced collisions, altered propagation behavior, and multi-component differential transport in terms of physical trends; its further contribution lies in the establishment of a component-resolved transport interpretation framework applicable to the Al–Ti/Ar binary system through ever-collided statistics, Ti-normalized mechanism partitioning, and analysis of the initial high-velocity range.

## 5. Conclusions

In this study, a kinetic-statistical approach was employed to investigate the propagation behavior and species-dependent transport characteristics of an Al_0.75_Ti_0.25_ plume in a low-pressure Ar background. The results show that, within the pressure range of 0.001–1 Pa, the number of particles that have experienced collisions increases markedly with increasing background pressure; at t = 0.56 μs, the cumulative number of particles that have undergone collisions increases approximately power-law with rising pressure, indicating that the influence of the background gas on plume propagation continues to strengthen. Meanwhile, across the entire pressure range and throughout 0.08–0.56 μs, the collision fraction of Ti remains consistently higher than that of Al, indicating persistent species-dependent differences during plume propagation.

Further analysis revealed that this discrepancy is related to the initial velocity distributions and kinetic energy distributions of the two types of particles. When velocities exceed approximately 4.37 km/s, the kinetic energy contribution from a single velocity bin for Ti exceeds that of Al; when velocities exceed approximately 5.22 km/s, the number of particles in a single velocity bin for Ti exceeds that of Al. Although particles in the high-velocity range account for only about 5.75% of the total particle count, they contribute approximately 25.9% of the total kinetic energy, with about 72% of this coming from Ti. These results indicate that Ti has a greater tendency to undergo collisions during the early stage of plume propagation, and that the observed transport differences arise not only from mass effects, but also from differences in the initial velocity and energy distributions.

Based on the degree of collision impact, this paper divides the pressure range into the near-free-flight region, the transition region, and the collision-affected region. The results show that within the 0.08–0.56 μs time range, the boundary pressure between the near-free-flight zone and the transition zone is approximately 0.097–0.100 Pa, while the boundary pressure between the transition zone and the collision-affected zone is approximately 0.494–0.500 Pa. The small overall variation indicates that pressure is the primary parameter controlling the plume propagation regime. These findings indicate that background pressure affects not only the collision intensity and propagation regime of the plume, but also the velocity, energy, and composition of particles reaching the substrate, thereby strongly influencing deposition efficiency, compositional uniformity, and final film quality. For Al–Ti binary alloy thin films, appropriately adjusting the background pressure helps control plume propagation behavior and optimize the coating process.

## Figures and Tables

**Table 1:** Numerical Conditions and Key Parameters.

Parameter	Value/Description
laser fluence F	8 J/cm^2^
Target atomic ratio	Al_0.75_Ti_0.25_
Background gas	Ar (inert background; chemical reactions are not taken into account)
Background temperature $T_{bg}$	300 K
Pressure $P_{bg}$	$\left\{0.001,0.003,0.01,0.03,0.1,0.3,1\right\}\;Pa$
Time bin t	$\left\{0.08,0.16,0.24,0.32,0.40,0.48,0.56\right\}\;\mu s$
bin	∆r=5 μm
Collision statistical cross section	ever-collided (A collision is counted if it has occurred at least once by time t)

**Table 2:** Full power-law fitting parameters for Ar, Al, and Ti over 0.08–0.56 μs (log–log fit).

Time/μs	Species	$\alpha_{s}$	$\log_{10}C_{s}$	$R^{2}$
0.08	Ar	1.012552	8.287991	0.999906
0.08	Al	1.105616	8.117459	0.995005
0.08	Ti	1.073421	8.578616	0.997187
0.16	Ar	1.007025	9.144746	0.999972
0.16	Al	1.068337	9.229536	0.997812
0.16	Ti	1.060020	9.372935	0.997849
0.24	Ar	1.004932	9.650015	0.999985
0.24	Al	1.059446	9.819395	0.997949
0.24	Ti	1.051075	9.819695	0.998293
0.32	Ar	1.004064	10.008102	0.999989
0.32	Al	1.054964	10.215581	0.997909
0.32	Ti	1.046265	10.135187	0.998493
0.40	Ar	1.003730	10.284494	0.999990
0.40	Al	1.048448	10.507690	0.998337
0.40	Ti	1.043224	10.379829	0.998564
0.48	Ar	1.003594	10.508337	0.999989
0.48	Al	1.044148	10.739507	0.998582
0.48	Ti	1.040717	10.579365	0.998604
0.56	Ar	1.003465	10.695446	0.999988
0.56	Al	1.041730	10.931513	0.998670
0.56	Ti	1.038531	10.747860	0.998626

Note: The fitted form is $N_{collided}^{\left(s\right)}\left(P\right)=C_{s}P^{\alpha_{s}}$ . The table shows $\alpha_{s}$ versus $\log_{10}C_{s}$ and the fitted $R^{2}$. The table lists the fitted exponent $\alpha_{s}$, the logarithmic prefactor $\log_{10}C_{s}$, and the fitting quality $R^{2}$ for Ar, Al, and Ti over the full 0.08–0.56 μs time window.

**Table 3:** Summary of cross-pressure statistics at t = 0.56 μs.

P/Pa	Al_collided_	Ti_collided_	Al_coll-frac_	Ti_coll-frac_
0.001	5.43 × 10^7^	3.64 × 10^7^	3.78 × 10^−7^	7.25 × 10^−7^
0.003	2.16 × 10^8^	1.43 × 10^8^	1.50 × 10^−6^	2.86 × 10^−6^
0.01	7.77 × 10^8^	5.17 × 10^8^	5.42 × 10^−6^	1.03 × 10^−5^
0.03	2.37 × 10^9^	1.58 × 10^9^	1.65 × 10^−5^	3.15 × 10^−5^
0.1	7.95 × 10^9^	5.28 × 10^9^	5.54 × 10^−5^	1.05 × 10^−4^
0.3	2.38 × 10^10^	1.58 × 10^10^	1.66 × 10^−4^	3.14 × 10^−4^
1	7.91 × 10^10^	5.14 × 10^10^	5.51 × 10^−4^	1.02 × 10^−3^

**Table 4:** Ti Criterion Mechanism Threshold Pressures.

Time/μs	$P_{1}\left(\Phi_{Ti}=0.1\right)$	$P_{2}\left(\Phi_{Ti}=0.5\right)$
0.08	0.100372	0.499967
0.16	0.099825	0.499019
0.24	0.099325	0.497969
0.32	0.098819	0.496893
0.40	0.098313	0.495795
0.48	0.097803	0.494672
0.56	0.097284	0.493532

## Data Availability

The original contributions presented in this study are included in the article/Supplementary Materials. Further inquiries can be directed to the corresponding author.

## Supplementary Materials

The following supporting information can be downloaded at https://www.mdpi.com/article/10.3390/ma19091904/s1: Table S1. Definitions of Raw Data Columns and Derived Statistics. Table S2. Wichtige statistische Kennzahlen des Anfangsgeschwindigkeitsspektrums. Table S3. Evolution of the collision fraction over time at various pressures.

## Abbreviations

The following abbreviations are used in this manuscript:

PLA	Pulsed Laser Ablation
MB	Maxwell–Boltzmann
LIBS	Laser-Induced Breakdown Spectroscopy
ICCD	Intensified Charge-Coupled Device
