# Supplementary material for: Collision Mechanisms of Particles in the Al–Ti Plasma Plume Induced by Pulsed Laser Ablation
Source: Materials (Basel). 2026 May 6;19(9):1904. doi: 10.3390/ma19091904 (PMC13165270; doi:10.3390/ma19091904)
Supplement: Supplementary file 1 [file materials-19-01904-s001.zip › materials-4284894-supplementary.pdf]

Table S1. Definitions of Raw Data Columns and Derived Statistics

Category	Symbol/Column	Meaning (Unit)
Original output（distance bin）	θ	Perspective（deg）
	t	Time（μs）
	r	Distance（m）
	$N^{Ar}(t,r)$	Number of Ar particles in the bin
	$N^{Al}(t,r)$	Number of Al particles in the bin
	$N^{Ti}(t,r)$	Number of Ti particles in the bin
	$N_{coll}^{Ar}(t,r)$	Number of Ar particles that have collided within the bin (⩾1)
	$N_{coll}^{Al}(t,r)$	Number of Al particles that have collided within the bin (⩾1)
	$N_{coll}^{Ti}(t,r)$	Number of Ti particles that have collided within the bin (⩾1)
Derivative	$N_{collided}^{(s)}(t;P)=\sum_r N_{coll}^{(s)}(t,r)$	The cumulative number of particles that have ever collided with species s (ever-collided counts)
	$f_{coll}^{(s)}(t;P)=\frac{\sum_r N_{coll}^{(s)}(t,r)}{\sum_r N^{(s)}(t,r)}$	Collision Rate (Percentage of particles that have collided)
Ti-criterion normalization	$\Phi_{Ti}(P,t)=\frac{N_{collided}^{Ti}(t;P)}{N_{collided}^{Ti}(t;1Pa)}$	Dimensionless metrics

index		for mechanistic partitioning (normalized to 1 Pa at a given time)
Mechanical boundary pressure	$P_1(t)(\Phi_{Ti} = 0.1);\ P_2(t)(\Phi_{Ti} = 0.5)$	Crossover / regime-boundary pressures

Table S2. Wichtige statistische Kennzahlen des Anfangsgeschwindigkeitsspektrums

Indicators	Ti	Al
N_{total}(count)	5.231×10^{15}	1.619×10^{16}
v_{mean} (m/s)	3229.506	2544.505
v_{rms} (m/s)	3623.585	2832.571
v_{median} (m/s)	3031.244	2434.934
$v_{90\%}$ (m/s)	5515.871	4174.172
$v_{99\%}$ (m/s)	7354.494	6037.642
v_{E} energy-crossover (m/s)	4372.942	4372.942
v_{N} count-crossover (m/s)	5217.715	5217.715
Tail number fraction ($v \geq v_{\mathrm{N}}$)	0.057501	0.057501
Tail energy fraction ($v \geq v_{\mathrm{N}}$)	0.259374	0.259374
Ti share of tail energy	0.719664	0.719664

Note: The statistics are based on speed-binned counts (a discrete distribution weighted by the bin center speed). Cross-thresholds: v_{E} is the minimum speed at which the kinetic energy contribution Ti exceeds Al; v_{N} is the minimum speed at which the count Ti exceeds Al. Tail definition: $v \geq v_{\mathrm{N}}$.

Table S3. Evolution of the collision fraction over time at various pressures

Table S3 (a). Ar: $f_{coll}^{Ar}(t;P)$

Time/us	P/0.001 Pa	P/0.003 Pa	P/0.01 Pa	P/0.03 Pa	P/0.1 Pa	P/0.3 Pa	P/1 Pa
0.08	1.385×10^{-4}	1.486×10^{-4}	1.526×10^{-4}	1.538×10^{-4}	1.542×10^{-4}	1.543×10^{-4}	1.542×10^{-4}
0.16	4.684×10^{-4}	5.174×10^{-4}	5.364×10^{-4}	5.422×10^{-4}	5.442×10^{-4}	5.448×10^{-4}	5.444×10^{-4}
0.24	1.043×10^{-3}	1.174×10^{-3}	1.225×10^{-3}	1.240×10^{-3}	1.246×10^{-3}	1.248×10^{-3}	1.247×10^{-3}
0.32	1.84×10^{-3}	2.108×10^{-3}	2.210×10^{-3}	2.241×10^{-3}	2.252×10^{-3}	2.256×10^{-3}	2.254×10^{-3}
0.4	2.817×10^{-3}	3.262×10^{-3}	3.436×10^{-3}	3.489×10^{-3}	3.508×10^{-3}	3.515×10^{-3}	3.511×10^{-3}
0.48	3.937×10^{-3}	4.610×10^{-3}	4.874×10^{-3}	4.955×10^{-3}	4.984×10^{-3}	4.994×10^{-3}	4.989×10^{-3}
0.56	3.876×10^{-2}	3.956×10^{-2}	3.997×10^{-2}	4.008×10^{-2}	4.010×10^{-2}	4.011×10^{-2}	3.986×10^{-2}

Table S3 (b). Al: $f_{coll}^{Al}(t;P)$

Time/us	P/0.001 Pa	P/0.003 Pa	P/0.01 Pa	P/0.03 Pa	P/0.1 Pa	P/0.3 Pa	P/1 Pa
0.08	1.607×10^{-9}	6.405×10^{-9}	2.300×10^{-8}	6.996×10^{-8}	2.336×10^{-7}	7.021×10^{-7}	2.328×10^{-6}
0.16	1.199×10^{-8}	4.698×10^{-8}	1.680×10^{-7}	5.094×10^{-7}	1.701×10^{-6}	5.105×10^{-6}	1.701×10^{-5}
0.24	4.263×10^{-8}	1.659×10^{-7}	5.950×10^{-7}	1.802×10^{-6}	6.009×10^{-6}	1.802×10^{-5}	6.009×10^{-5}
0.32	1.031×10^{-7}	4.008×10^{-7}	1.436×10^{-6}	4.352×10^{-6}	1.450×10^{-5}	4.352×10^{-5}	1.450×10^{-4}

0.4	2.045×10^{-7}	7.936×10^{-7}	2.842×10^{-6}	8.613×10^{-6}	2.871×10^{-5}	8.613×10^{-5}	2.871×10^{-4}
0.48	3.556×10^{-7}	1.377×10^{-6}	4.934×10^{-6}	1.495×10^{-05}	4.985×10^{-5}	1.495×10^{-4}	4.985×10^{-4}
0.56	3.783×10^{-7}	1.504×10^{-6}	5.415×10^{-6}	1.654×10^{-05}	5.541×10^{-5}	1.661×10^{-4}	5.511×10^{-4}

Table S3 (c). Ti： $f_{coll}^{Ti}(t;P)$

Time/us	P/0.001 Pa	P/0.003 Pa	P/0.01 Pa	P/0.03 Pa	P/0.1 Pa	P/0.3 Pa	P/1 Pa
0.08	3.408×10^{-9}	1.334×10^{-8}	4.775×10^{-8}	1.450×10^{-7}	4.859×10^{-7}	1.450×10^{-6}	4.859×10^{-6}
0.16	2.563×10^{-8}	9.842×10^{-8}	3.522×10^{-7}	1.070×10^{-6}	3.571×10^{-6}	1.070×10^{-5}	3.571×10^{-5}
0.24	9.216×10^{-8}	3.522×10^{-7}	1.258×10^{-6}	3.817×10^{-6}	1.271×10^{-5}	3.817×10^{-5}	1.271×10^{-4}
0.32	2.242×10^{-7}	8.578×10^{-7}	3.062×10^{-6}	9.287×10^{-6}	3.096×10^{-5}	9.287×10^{-5}	3.096×10^{-4}
0.4	4.461×10^{-7}	1.706×10^{-6}	6.090×10^{-6}	1.848×10^{-5}	6.157×10^{-5}	1.848×10^{-4}	6.157×10^{-4}
0.48	7.772×10^{-7}	2.984×10^{-6}	1.064×10^{-5}	3.227×10^{-5}	1.075×10^{-4}	3.227×10^{-4}	1.075×10^{-3}
0.56	7.252×10^{-7}	2.858×10^{-6}	1.031×10^{-5}	3.149×10^{-5}	1.053×10^{-4}	3.143×10^{-4}	1.024×10^{-3}
